# Role of palliative radiotherapy in bleeding control in patients with unresectable advanced gastric cancer

**DOI:** 10.1186/s12885-021-08145-4

**Published:** 2021-04-15

**Authors:** Jesang Yu, Jinhong Jung, Sook Ryun Park, Min-Hee Ryu, Jin-hong Park, Jong Hoon Kim, Sang Min Yoon

**Affiliations:** 1 Department of Radiation Oncology, Asan Medical Center, University of Ulsan College of Medicine, 88, Olympic-ro 43-gil, Songpa-gu, Seoul, 05505 Republic of Korea; 2 Department of Oncology, Asan Medical Center, University of Ulsan College of Medicine, Seoul, Republic of Korea

**Keywords:** Radiotherapy, Chemotherapy, Stomach neoplasm, Hemorrhage, Hemostasis

## Abstract

**Background:**

This study analyzed the clinical results of palliative radiotherapy for bleeding control in patients with unresectable advanced gastric cancer.

**Methods:**

We retrospectively reviewed the medical records of patients who met the following inclusion criteria between January 2002 and June 2018: histologically proven gastric cancer, gastric tumor bleeding confirmed by upper gastrointestinal endoscopy, and palliative radiotherapy performed for hemostasis. The median radiotherapy dose was 30 Gy, with a daily dose ranging from 1.8 to 3 Gy.

**Results:**

Sixty-one patients were included in this analysis. The study population was predominantly male (72.1%), with a median age of 62 years (range: 32–92). The median baseline hemoglobin level was 7.1 g/dL, and the most common presenting symptom of gastric tumor bleeding was melena (85.2%). Bleeding control was achieved in 54 (88.5%) patients. The median levels of hemoglobin at 1, 2, and 3 months after completion of radiotherapy were 10.1 g/dL, 10.2 g/dL, and 10.4 g/dL, respectively; these values were significantly different from that before radiotherapy (7.1 g/dL; *p* < 0.001). The median overall survival was 4.8 months. Among the 54 patients who achieved bleeding control after radiotherapy, 19 (35.2%) experienced re-bleeding during the follow-up period. The median time to re-bleeding was 6.0 months. Multivariate analysis demonstrated that a higher radiation dose (*p* = 0.007) and additional chemotherapy after radiotherapy (*p* = 0.004) were significant factors for prolonging the time to re-bleeding.

**Conclusions:**

Tumor bleeding was adequately controlled by radiotherapy in patients with unresectable advanced gastric cancer.

## Background

Gastric cancer is the fifth most common malignancy and the third leading cause of cancer-related mortality worldwide [1]. Although its incidence has started to decrease recently, gastric cancer is still the most commonly diagnosed cancer and the third most common cause of cancer-related deaths in Korea [2]. The standard therapy for patients with localized gastric cancer is gastrectomy with extended lymphadenectomy with or without perioperative chemotherapy [3, 4]. However, some patients are diagnosed at an unresectable stage of advanced gastric cancer, despite the presence of a thorough surveillance program for early detection. Patients with advanced disease usually experience local symptoms, including pain, bleeding, dysphagia, vomiting, and finally malnutrition, all of which can hinder the patients’ quality of life (QoL) [5]. Among these symptoms, gastric bleeding due to uncontrolled tumor growth results in severe anemia, and could eventually cause a life-threatening condition [6]. Various treatment modalities, such as palliative surgery, endoscopic hemostasis, and angiographic intervention, have been applied to control the bleeding caused by advanced gastric cancer [6–10]; however, these therapies are not effective in patients with very large infiltrating gastric masses or chronic oozing type of bleeding. Therefore, it is necessary to identify other therapeutic modalities to improve the QoL of these patients.

External beam radiotherapy has not been used as a primary treatment modality for gastric cancer; however, some studies have evaluated the role of palliative radiotherapy on hemostasis in patients with advanced gastric cancer and reported bleeding control rates that ranged from 53 to 80% [11–17]. Unfortunately, most of these series have limitations such as small study populations, use of outdated radiotherapy techniques, and lack of sufficient follow-up data after radiotherapy. In addition, hemostasis or re-bleeding is not consistently defined among these studies. Further research with reasonable study endpoints is necessary to understand the role of radiotherapy in these clinical conditions. Thus, we conducted this study to analyze the clinical outcomes of patients with unresectable advanced gastric cancer who received palliative radiotherapy for bleeding control.

## Methods

### Study population

We retrospectively reviewed the medical records of patients with unresectable advanced gastric cancer who received palliative radiotherapy for bleeding control at Asan Medical Center (Seoul, Korea) between January 2002 and June 2018. The inclusion criteria were as follows: (a) histologically proven gastric cancer; (b) gastric tumor bleeding confirmed by upper gastrointestinal endoscopy; and (c) palliative radiotherapy performed for hemostasis. Patients who received radiotherapy for alleviating gastric obstruction or pain without gastric tumor bleeding were excluded. This study was approved by the Institutional Review Board of Asan Medical Center (#2019–0056), and the requirement for informed consent was waived because of the retrospective nature of the present study. This study was conducted in accordance with the Declaration of Helsinki.

### Radiotherapy

Patients were immobilized in a supine position with a vacuum cushion, and free-breathing computed tomography (CT) with intravenous contrast agents was performed. Starting October 2015, 4-dimensional CT (GE LightSpeed RT 16; GE Health care, Waukesha, WI, USA) was routinely performed using a Real-time Position Management respiratory gating system (Varian Medical Systems, Palo Alto, CA, USA) for the analysis of the patients’ breathing pattern.

The gross tumor volume (GTV) was defined as a fungating mass or infiltrative gastric wall thickening with enhancement on planning CT. Position emission tomography-CT was also used to define the GTV, when available. The clinical target volume was equal to the GTV. Before 4-dimensional CT became available, the planning target volume (PTV) was added with a 1–2-cm margin from the GTV, in consideration of set-up uncertainty and respiratory motion. While using 4-dimensional CT, the internal target volume (ITV) was determined as the sum of individual GTVs as defined by the 10 respiratory phases. Then, the PTV was added with a 1-cm margin from the ITV. To reduce inter-fractional uncertainty, patients were required to fast for 4 h before each radiotherapy.

Treatment planning was performed using multiple coplanar or non-coplanar beams using a 3-dimensional radiotherapy planning system (Eclipse; Varian Medical Systems). Radiotherapy was delivered via a 3-dimensional conformal radiotherapy technique with free breathing using a linear accelerator (Varian Medical Systems).

### Other procedures

Upper gastrointestinal endoscopy was performed before radiotherapy for the diagnosis of tumor bleeding and/or emergency hemostasis. Transcatheter arterial embolization was also performed for the management of active bleeding in selected patients. Packed red blood cells were transfused before or at an early phase of radiotherapy for the correction of anemia at the discretion of the physician. Palliative chemotherapy for advanced gastric cancer was administered before the event of tumor bleeding and after bleeding control with radiotherapy to patients with good performance status.

### Evaluation

Regular examinations including those for symptoms and complaints, physical examinations, complete blood counts, and biochemical profiles were performed weekly during radiotherapy. Medical history taking, physical examinations, complete blood counts, biochemical profiles, and abdominopelvic CT were performed regularly at 2–3-month intervals after radiotherapy in general. However, the number of examinations was increased at the discretion of the physicians in cases of unexpected events. Toxicity induced by radiotherapy was graded according to the Common Terminology Criteria for Adverse Events.

To evaluate palliative efficacy, the lowest levels of hemoglobin between pre-radiotherapy hemostasis procedures and radiotherapy were regarded as the baseline hemoglobin levels. We defined ‘bleeding control’ as a status that fulfills the following conditions: (a) the clinical symptoms associated with gastric bleeding, such as melena or hematemesis, completely disappeared; (b) hemoglobin levels after radiotherapy did not decline compared with those at baseline; and (c) no further transfusions were needed. Re-bleeding was defined as follows: (a) reappearance of clinical symptoms and/or (b) necessity of additional transfusions due to a decline in hemoglobin levels in the absence of symptoms related to gastric bleeding. However, patients receiving transfusions due to other definite bleeding foci with no gastric bleeding-related symptoms were not considered to have re-bleeding events. We also did not consider decreases in hemoglobin levels associated with myelosuppression after additional chemotherapy as re-bleeding. Because blood transfusion was not performed at regular intervals, the transfusion volume was converted into an average daily value to compare between the amount of blood transfused before and after radiotherapy.

### Statistical analysis

The differences in hemoglobin levels and the amount of blood transfused before and after radiotherapy were analyzed using the paired t-test. Overall survival (OS) was estimated from the date of radiotherapy initiation to the date of death or the last follow-up. The cumulative incidence of re-bleeding after radiotherapy was also estimated from the start of radiotherapy to the date of the re-bleeding event, according to the abovementioned definitions. The probability of cumulative survival was calculated using the Kaplan–Meier method. Cox proportional hazard models were generated to describe the associations of covariates with the cumulative incidence of re-bleeding. A *p* value < 0.05 was considered statistically significant. All statistical analyses were performed using R software (version 3.6.3, R Foundation Inc.; http://cran.r-project.org/ and web-r.org).

## Results

### Patient characteristics

**Fig. 1:**
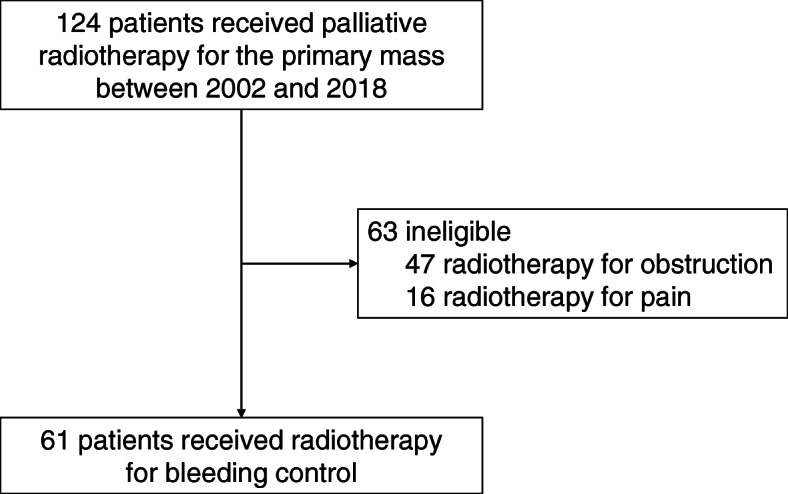
Flow diagram of the inclusion criteria used in the study population

We identified 124 patients who received palliative radiotherapy for unresectable gastric cancer during the study period. Among them, 47 and 16 patients received palliative radiotherapy for the treatment of gastrointestinal obstructions and pain management, respectively. Therefore, a total of 61 patients were included in this analysis (Fig. 1). The patient characteristics are shown in Table 1. The study population mainly consisted of male patients (72.1%); the median age was 62 years (range: 32–92). Almost two-thirds of the patients showed a poor performance status before radiotherapy. All patients were considered as unresectable or medically inoperable status when considering the palliative radiotherapy: 41 patients had distant metastases, 6 patients had unresectable locally advanced gastric cancer, and 13 patients had severe comorbidities including heart diseases (coronary artery diseases, atrial fibrillation, or aortic aneurysm), stroke, or renal/pulmonary dysfunction. No patients had received anticoagulation therapy for their comorbidities within 1 month prior to, during, and after radiotherapy. The median baseline hemoglobin level was 7.1 g/dL (range: 3.3–10.4), and the most common (85.2%) presenting symptom of gastric tumor bleeding was melena.

**Table 1:** Summary of the patient characteristics and treatments

Variables	No. of patients (%) or Median (range)
Sex	
Male	44 (72.1)
Female	17 (27.9)
Age (years)	62 (32–92)
ECOG performance status before radiotherapy	
< 2	19 (31.1)
≥ 2	42 (68.9)
Disease status before radiotherapy	
Locally advanced disease	20 (32.8)
Combined locally advanced and metastatic disease	41 (67.2)
Pathology	
Tubular adenocarcinoma	56 (91.8)
Signet ring cell carcinoma	5 (8.2)
Location	
Proximal	10 (16.4)
Body	25 (41.0)
Distal	26 (42.6)
Baseline hemoglobin level (g/dL)	7.1 (3.3–10.4)
Initial symptoms^a^	
Melena	52 (85.2)
Hematemesis	20 (32.8)
Endoscopic hemostasis before radiotherapy	
Yes	30 (49.2)
No	31 (50.8)
Angiographic intervention before radiotherapy	
Yes	10 (16.4)
No	51 (83.6)
Radiotherapy dose (Gy)	30 (12.5–50)
Previous chemotherapy before gastric bleeding	
Yes	50 (82.0)
No	11 (18.0)
Additional chemotherapy after radiotherapy	
Yes	30 (49.2)
No	31 (50.8)

*ECOG* Eastern Cooperative Oncology Group

^a^Percentages add up to > 100% because 11 patients had both symptoms at initial presentation.

### Summary of treatments

After confirmation of gastric bleeding by endoscopy, 30 (49.2%) patients underwent endoscopic hemostasis procedures using fibrin glue, hemostatic forceps, argon plasma coagulation, or epinephrine spray before radiotherapy. Transcatheter arterial embolization was performed in 10 patients to control active bleeding (Table 1). Among these, three patients received both endoscopic and angiographic interventions for initial bleeding control. All interventions were performed as an emergency procedure for patients whose hemoglobin level was very low due to severe bleeding from gastric cancer. However, because follow-up endoscopic evaluations showed that the bleedings had not been completely controlled, palliative radiotherapies were requested.

Planned radiotherapy was completed in 51 (83.6%) patients; 10 patients could not finish radiotherapy as planned because of worsening of their performance status (*n* = 7), refusal to continue radiotherapy (*n* = 2), or resumption of palliative chemotherapy after rapid improvement of symptoms (*n* = 1). The median radiotherapy dose was 30 Gy (range: 12.5–50) with a daily dose of 1.8–3 Gy. The median biologically effective dose (BED, α/β = 10) was 39 Gy_10_ (range: 16–60).

Most patients (82.0%) received palliative chemotherapy before tumor bleeding events; however, these patients stopped receiving chemotherapy during interventions for bleeding control and palliative radiotherapy. After tumor bleeding was controlled by radiotherapy, 30 (49.2%) patients were treated with palliative chemotherapy for gastric cancer. Various chemotherapeutic agents, such as capecitabine, 5-fluorouracil, oxaliplatin, irinotecan, S-1, paclitaxel, docetaxel, and cisplatin, were used in single or combination regimens.

### Bleeding control after radiotherapy

**Fig. 2:**
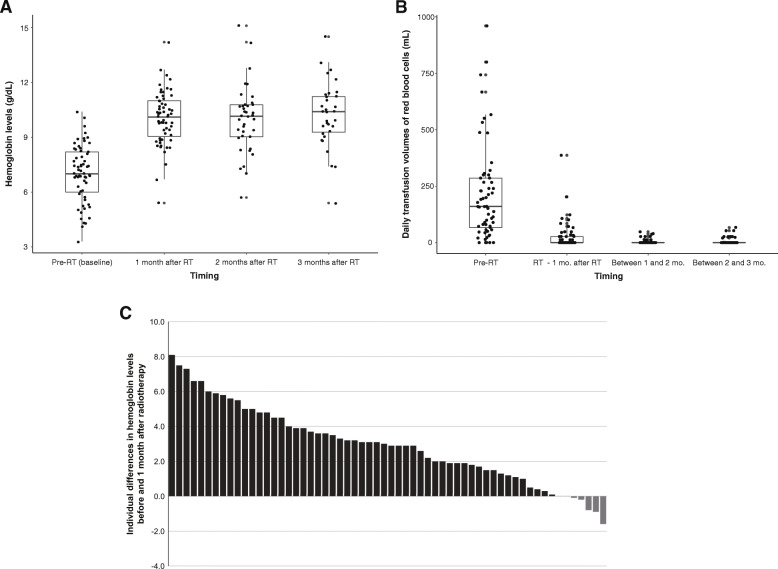
Bleeding control after palliative radiotherapy. **a** Hemoglobin levels before and after radiotherapy, (**b**) Daily transfusion volumes of red blood cells before and after radiotherapy, and (**c**) Individual differences in hemoglobin levels before and 1 month after radiotherapy

Bleeding control was achieved in 54 (88.5%) patients after radiotherapy. The median time from the start of radiotherapy to bleeding control was 13 days (range: 1–68). The median levels of hemoglobin at 1, 2, and 3 months after radiotherapy completion were 10.1 g/dL (range: 5.4–14.2), 10.2 g/dL (range: 5.7–15.1), and 10.4 g/dL (range: 5.4–14.5), respectively; these values were significantly higher than that before radiotherapy (*p* < 0.001, Fig. 2a). The average daily blood transfusion volume during the period between the determination of the baseline hemoglobin level and radiotherapy initiation was 217 mL; this average volume decreased significantly after radiotherapy: 108 mL during the first month, 4 mL during the second month, and 6 mL during the third month (*p* < 0.001, Fig. 2b). Figure 2c shows the differences between the baseline hemoglobin level before radiotherapy and hemoglobin levels 1 month after radiotherapy. Most patients (86.9%) showed increased hemoglobin levels 1 month after radiotherapy (range: 0.1–8.1 g/dL).

### Survival outcomes and follow-up results

**Fig. 3:**
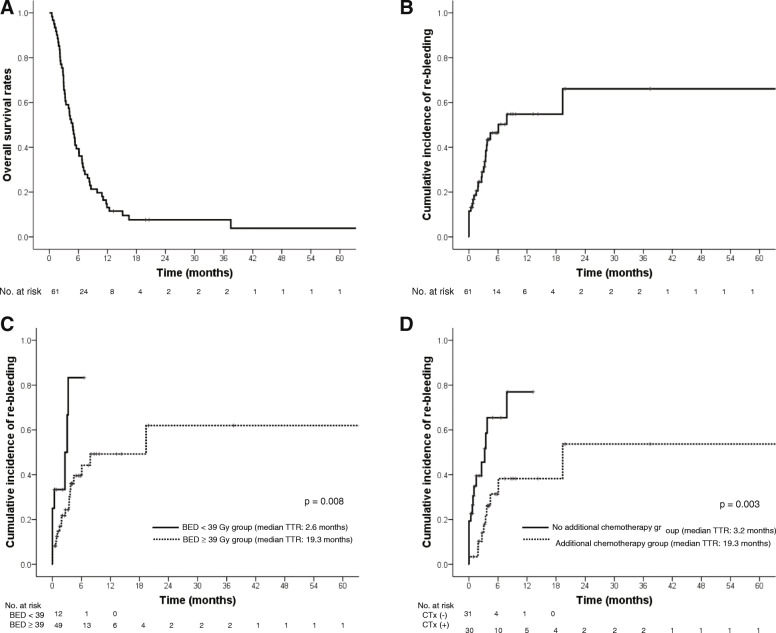
Survival outcomes after palliative radiotherapy. **a** Overall survival rates, (**b**) Time to re-bleeding, (**c**) Time to re-bleeding according to biologically effective dose_10_ (< 39 Gy vs. ≥_39 Gy), and (**d**) Time to re-bleeding according to additional chemotherapy after radiotherapy

The median follow-up period was 3.9 months (range: 0.3–103.1). At the time of analysis, 4 (6.6%) of 61 patients were alive, and the median OS was 4.8 months (95% confidence interval [CI]: 3.7–5.9) (Fig. 3a). Among the 54 patients who achieved bleeding control after radiotherapy, 19 (35.2%) patients experienced re-bleeding during the follow-up period. The median time to re-bleeding was 6.0 months (Fig. 3b). High BED_10_ (hazard ratio [HR] = 0.871; 95% CI: 0.788–0.963; *p* = 0.007) and use of chemotherapy after radiotherapy (HR = 0.276; 95% CI, 0.114–0.670; *p* = 0.004) were statistically significant factors for prolonging the time to re-bleeding in multivariate analysis (Table 2, Fig. 3c, and d).

**Table 2:** Prognostic factors for the time to re-bleeding

Variables	Univariate analysis		Multivariate analysis	
	Hazard ratio (95% CI)	*p* value	Hazard ratio (95% CI)	*p* value
Age	0.977 (0.948–1.007)	0.129		
ECOG performance status (< 2)	1.599 (0.669–3.820)	0.291		
Hematemesis (no)	1.544 (0.679–3.509)	0.300		
Hemostasis interventions before radiotherapy (no)	1.542 (0.678–3.504)	0.301		
Baseline hemoglobin level	1.052 (0.824–1.342)	0.684		
Amount of transfusion before radiotherapy	1.020 (0.973–1.070)	0.409		
Biologically effective dose	0.919 (0.875–0.966)	0.001	0.871 (0.788–0.963)	0.007
Additional chemotherapy after radiotherapy (no)	0.303 (0.132–0.697)	0.005	0.276 (0.114–0.670)	0.004

*CI* Confidence interval; *ECOG* Eastern Cooperative Oncology Group

Values in parentheses were set as the reference.

### Treatment-related toxicity

One (1.6%) patient experienced acute grade 3 intractable nausea during radiotherapy and stopped receiving radiotherapy early after receiving 18 Gy. Other constitutional symptoms, including anorexia, nausea, fatigue, and abdominal pain, which were controlled by medication, were mostly grade 1 or 2. No grade ≥ 4 acute toxicity or other late toxicities were observed during or after radiotherapy.

## Discussion

Our study demonstrated that radiotherapy provided a high rate of hemostasis for tumor bleeding in patients with unresectable advanced gastric cancer, although re-bleeding was observed in almost one-third of the patients. To reduce the number of re-bleeding events, a higher radiation dose and additional palliative chemotherapy after bleeding control would be necessary according to the patients’ general condition. In addition, this study provided meaningful information in that we performed all treatment planning using up-to-date radiotherapy techniques including 4 dimensional-CT; moreover, in order to aptly represent the palliative efficacy of radiotherapy in patients with poor prognosis, our study provided the kinetics of hemostasis before and after radiotherapy by using practical endpoints in terms of bleeding control and re-bleeding.

The most common initial hemostatic procedure in patients with advanced gastric cancer is endoscopic hemostasis using various materials [8, 18, 19]. However, re-bleeding is frequently observed even after initial hemostasis [8, 18, 19], and the rate of re-bleeding tends to be higher in patients with cancer bleeding than in those with benign ulcer bleeding [9, 20]. Furthermore, the efficacy of endoscopic procedures is limited by the location of the lesion, lesion size, and type of bleeding (massive and/or diffuse) [7, 10, 21]. Because tumor bleeding from advanced gastric cancer usually occurs in cases involving uncontrolled infiltrative gastric masses, endoscopic procedures may not be effective for achieving long-term bleeding control. Transcatheter arterial embolization is another treatment option, and it is often used after the failure of endoscopic hemostasis [10, 22]. However, it has potential technical limitations associated with the following factors: difficulty in identifying the exact bleeding site, no definitive arterial bleeding, and lack of intervention skills depending on the physician [7, 23]. Therefore, transcatheter arterial embolization also has limited efficacy in patients with gastric cancer bleeding [7].

In the present study, the initial bleeding control rate was as high as 88.5%, and this result was similar to those of previously reported studies [11–17]. According to these promising outcomes, we believe that radiotherapy would be a good treatment modality for gastric cancer bleeding. The high rate of initial bleeding control might have been derived from the direct effect of radiotherapy in regressing the uncontrolled tumor burden [11]. There has been no definite conclusion regarding the relationship between radiation dose and the initial bleeding control rate. Lee et al. reported that a BED_10_ ≥ 36 Gy was significantly associated with bleeding control after radiotherapy [11]. In contrast, Tey et al. demonstrated that there were no differences in the response rate between low (≤ 39 Gy) and high (> 39 Gy) BED_10_ [14]. Moreover, Chaw et al. [13] and Kawabata et al. [24] indicated similar palliative effects with relatively low doses of radiotherapy, compared to those used in previous studies, in patients with advanced gastric cancer.

Duration of hemostasis is also an important endpoint for the QoL of these patients. Our results demonstrated that the median time to re-bleeding was significantly longer in patients treated with higher BED_10_ (≥ 39 Gy) than in those receiving lower BED_10_ (19.3 months vs. 2.6 months; *p* = 0.008). Tey et al. reported that recurrent bleeding occurred more frequently in patients who received a BED_10_ ≤ 39 Gy than in those who received a BED_10_ > 39 Gy, although the difference was not statistically significant (36.1% vs. 22.2%; *p* = 0.78) [14]. Hashimoto et al. showed similar outcomes for BED_10_ ≥ 50 Gy, which was significantly correlated with treatment success (*p* = 0.040) in 19 patients treated with radiotherapy for hemorrhage of unresectable gastric cancer [17]. Although few studies have evaluated hemostasis duration in this setting due to the difficulty of conducting regular follow-ups in these patients, the above results showed that higher prescribed doses might delay the recurrence of symptoms after radiotherapy. Further studies regarding both the initial and long-term palliative effects of radiotherapy are necessary to define the optimal prescribed dose according to the prognosis of these patients.

Chemotherapy has been considered as the main therapeutic option for patients with advanced gastric cancer [25–28]; however, local symptoms such as bleeding, dysphagia, and vomiting can cause the interruption of palliative chemotherapy. Therefore, palliative treatments to relieve these symptoms may be important to provide an opportunity to resume or maintain chemotherapy. Our study showed that almost half of the patients (49.2%) could receive chemotherapy after radiotherapy, and this treatment also significantly reduced the risk of re-bleeding on multivariate analysis (HR = 0.276; 95% CI, 0.114–0.670; *p* = 0.004). According to the present results, palliative radiotherapy followed by additional chemotherapy could improve clinical outcomes in selected patients.

This study has the following limitations: first, the results may have a potential bias because this is a retrospective study with a small number of patients; second, although we tried to thoroughly review all follow-up results after radiotherapy, insufficient data were found for some patients because of their short-term survival after treatment. Therefore, patients with good performance status might receive more aggressive treatments, including a higher radiation dose and additional chemotherapy. Finally, although we used more reasonable and practical endpoints regarding hemostasis and re-bleeding after radiotherapy, all these endpoints could also have been affected by the accompanying chronic diseases and/or other procedures such as prior hemostasis by endoscopy, active transfusion, and further treatments after palliative radiotherapy. Hence, these results should be interpreted cautiously considering all these limitations. Nevertheless, this study is meaningful because it provides detailed statistical results in terms of re-bleeding events during follow-up, as well as in terms of the initial palliative effects after radiotherapy using modern radiotherapy techniques.

## Conclusions

Tumor bleeding was well controlled by radiotherapy in patients with unresectable advanced gastric cancer. Treatment decisions should be tailored according to the patients’ condition; a higher radiation dose and additional chemotherapy after radiotherapy could result in better treatment outcomes in patients with good performance status. Further studies are necessary to determine the optimal treatments for enhancing palliative effects in patients with tumor bleeding from advanced gastric cancer.

## Data Availability

The datasets generated and/or analysed during the current study are not publicly available due to the Personal Information Protection Act, but are available from the corresponding author upon reasonable request.

## Abbreviations

BED: Biologically effective dose
CI: Confidence interval
CT: Computed tomography
GTV: Gross tumor volume
HR: Hazard ratio
ITV: Internal target volume
OS: Overall survival
PTV: Planning target volume
QoL: Quality of life
